# MicroRNA expression patterns and target prediction in multiple myeloma development and malignancy

**DOI:** 10.1007/s13258-017-0518-7

**Published:** 2017-02-09

**Authors:** Ivyna Pau Ni Bong, Ching Ching Ng, Puteri Baharuddin, Zubaidah Zakaria

**Affiliations:** 1 Haematology Unit, Cancer Research Centre, Institute for Medical Research, Jalan Pahang, 50588 Kuala Lumpur, Malaysia; 2 Faculty of Science, Institute of Biological Sciences, University of Malaya, 50603 Kuala Lumpur, Malaysia

**Keywords:** Multiple myeloma, MicroRNA, Microarray, MiRNA-mRNA integrative analysis, MiR-150, MiR-125b

## Abstract

Epigenetic changes have emerged as key causes in the development and progression of multiple myeloma (MM). In this study, global microRNA (miRNA) expression profiling were performed for 27 MM (19 specimens and 8 cell lines) and 3 normal controls by microarray. miRNA-targets were identified by integrating the miRNA expression profiles with mRNA expression profiles of the matched samples (unpublished data). Two miRNAs were selected for verification by RT-qPCR (miR-150-5p and miR-4430). A total of 1791 and 8 miRNAs were over-expressed and under-expressed, respectively in MM compared to the controls (fold change ≥2.0; p < 0.05). The miRNA-mRNA integrative analysis revealed inverse correlation between 5 putative target genes (*RAD54L, CCNA2, CYSLTR2, RASGRF2* and *HKDC1*) and 15 miRNAs (p < 0.05). Most of the differentially expressed miRNAs are involved in survival, proliferation, migration, invasion and drug resistance in MM. Some have never been described in association with MM (miR-33a, miR-9 and miR-211). Interestingly, our results revealed 2 miRNAs, which are closely related to B cell differentiation (miR-150 and miR-125b). For the first time, we suggest that miR-150 might be potential negative regulator for two critical cell cycle control genes, *RAD54L* and *CCNA2*, whereas miR-125b potentially target RAS and CysLT signaling proteins, namely RASGRF2 and CYSLTR2, respectively. This study has enhanced our understanding on the pathobiology of MM and opens up new avenues for future research in myelomagenesis.

**Electronic supplementary material:**

The online version of this article (doi:10.1007/s13258-017-0518-7) contains supplementary material, which is available to authorized users.

## Introduction

Multiple myeloma (MM) is a malignancy of B lymphocytes, characterised by clonal expansion of malignant plasma cells in the bone marrow and over-production of intact monoclonal immunoglobulin of a single type (M-protein) (Eslick and Talaulikar 2013). It is the second most common haematological malignancy in the world (De Mel et al. 2014). Despite central role of genomic changes, epigenetic changes such as DNA methylations, histone modifications and non-coding RNAs arise as crucial factors in the transformation and progression of this malignancy.

microRNAs (miRNAs) are a class of ~20–25 nucleotides small non-coding, double-stranded RNA molecules. The mature miRNAs bind to their targets (usually at the 3′ untranslated region) and regulate the target gene expression by translation repression or mRNA degradation (Bi and Chng 2014). miRNAs are important in regulating the gene expression essential for normal cellular functions, aberrant expression of miRNAs has been implicated in many human diseases and phenotypic variations including MM (Lorio and Crose 2012; Bi and Chng 2014). In cancer, miRNAs function as regulatory molecules that can act as either an oncomiR or a tumour suppressor (Bi and Chng 2014). Their abnormal expression causes tumour formation by disrupting mechanisms that controlling apoptosis, angiogenesis, cell proliferation, invasion, and other critical signaling pathways (Dimopoulos et al. 2014).

miRNA expression profiles have been shown to be a powerful tool in the identification of novel biomarkers for diagnosis, prognosis and treatment of MM. To date, the number of miRNA studies in MM is still limited and insufficient to delineate the actual molecular events underlying the pathogenesis of MM. In this study, global miRNA expression profiling was performed to identify potential miRNAs in the molecular pathogenesis of MM. Moreover, miRNA-targets were also examined by integrating the miRNA expression profiles with previously performed mRNA expression profiles of the matched samples (unpublished data) by databases prediction and inverse correlation analysis. This study has enhanced our understanding on the pathobiology of MM and opens up new avenues for future research in MM.

## Materials and methods

### Patients

Bone marrow aspirates or blood were collected from patients and healthy donors. Nineteen MM samples (MM1-MM19) and 3 healthy controls were recruited in this study (N1, N2 and N3). The age of these patients ranged between 28 and 74 years with a mean and median age of 57 and 61, respectively. Seventeen out of 19 patients were newly diagnosed MM cases while the remaining 2 were relapsed cases. Samples for new cases were collected from the patients before treatment. Only patients with plasma cell infiltration >10% were included in this study.

### Cell lines

Eight myeloma cell lines were used in this study. The RPMI-8226, U-266, MM.1S, and IM-9 MM cells were purchased from American Type Culture Collection (ATCC, USA). Myeloma cell lines KMS-28-BM, KMS-20, KMS-12-BM, and KMS-21-BM were obtained from Japanese Collection of Research Bioresources (JCRB) cell bank. Cells were cultured with RPMI1640 medium (ATCC) supplemented with 10–15% fetal bovine serum (Lonza) in an incubator at 37 °C with humidified 5% CO_2_. Cells were passaged every 3–4 days.

### Total RNA extraction

Total RNAs were extracted from samples by using Qiagen RNeasy mini kit following manufacturer’s protocol. On**-**column DNase digestion was performed with the RNase-free DNase set to eliminate DNA contamination during RNA purification (Qiagen DNase I). The quality of total RNAs was assessed using RNA Nano Chip in Agilent’s 2100 Bioanalyser. The RNA integrity number (RIN) was >8.0 for all the samples included in this study. The purity of the RNA samples (A260 nm/ A280 nm) was within the range of 1.80–2.10 as measured by NanoDrop ND-1000 UV–VIS spectrophotometer.

### miRNA microarray assay

Briefly, 100 ng of total RNAs were labeled using Agilent miRNA Complete Labeling and Hyb Kit (Agilent Technologies) following manufacturer’s standard processing recommendations. Labeled RNAs were purified with MicroBioSpin 6 Column (Bio-Rad) to wash off unincorporated dyes. Purified labeled RNAs were dried in a vacuum concentrator at low heat. Pellet containing labeled miRNAs were resuspended in hybridisation cocktails and hybridised to SurePrint Human miRNA Microarray, release 19.0, 8 × 60 K (Agilent Technologies), which contained probes for 2006 human miRNAs for 20 h at 55 °C with rotation. After incubation, microarray slide was washed and scanned with Agilent array scanner G2505C. Image was then analysed with Agilent Feature Extraction Software Version 10.7.3.1.

Arrays that passed QC criteria were proceeded for further analysis. All the miRNA profiles were analysed by GeneSpring software version 13.0. All the miRNA data were thresholded to 1 and normalised to 75th percentile. The entities were then filtered where at least 1 sample out of 30 samples have ‘present’ flag. Differentially expressed miRNAs in MM samples compared to the controls were determined by unpaired unequal variance *t* test. The Benjamin Hochberg false discovery rate multiple testing correction was applied. Entities were filtered at p-value cut-off 0.05 and fold change ≥2.0.

### miRNA-target prediction and inverse correlation analysis

Identification of miRNA-targets was carried out by integrating the miRNA expression profiles with previously performed mRNA expression profiles of the matched samples (unpublished data) using GeneSpring software version 13.0. Putative target genes of differentially expressed miRNAs were predicted by TargetScan at p < 0.05.

### Quantitative reverse transcription-PCR (RT-qPCR)

Due to limitation of samples, RT-qPCR was performed to verify the expression of 2 differentially expressed miRNAs (miR-150-5p and miR-4430). The cDNA synthesis was carried out using miScript Reverse Transcription Kit (Qiagen) in a final volume of 20 µl containing 1× miScript HiSpec Buffer, 1× miScript Nucleic Mix, miScript Reverse Transcriptase Mix and 500 ng of RNA template according to manufacturer’s instructions. miRNA specific primers, which were miR-150-5p (Cat. No.: HmiRQP0210) and miR-4430 (Cat. No.: HmiRQP2054) and internal control miRNA, RNU6 (Cat. No.: HmiRQP9001) were purchased from GeneCopoeia (USA). Quantification of miRNA expression levels were performed by using miScript SYBR Green PCR Kit (Qiagen) in Rotor-Gene Q 2-Plex (Qiagen, Hilden, Germany) according to manufacturer’s protocols.

The expression levels of the miRNAs were verified in 20–25 MM samples depending on the availability of the samples. Two normal controls were used in each assay. All the RT-qPCR reactions were carried out in duplicates. The Ct values were normalised against internal controls and the fold difference of expression levels were calculated through relative quantification using ${{2}^{-\Delta \Delta {{\text{C}}_{\text{t}}}}}$ method. The significance level of MM and control groups was determined by student’s t-test.

### Data availability

The miRNA microarray data generated in this study are available in the NCBI Gene Expression Omnibus (GEO) as series accession identifier GSE73048.

## Results

### Differentially expressed miRNAs in MM

A total of 1799 miRNAs were differentially expressed by ≥2.0 fold change in MM samples compared to controls at p < 0.05. Out of 1799 miRNAs, 1791 and 8 miRNAs were over-expressed and under-expressed, respectively in MM. The miRNAs were more frequently up-regulated rather than down-regulated in MM, which is consistent with microarray findings reported by Yusnita et al. (2012), Zhou et al. (2010) and Chi et al. (2011). The 8 under-expressed miRNAs were identified as miR-342-5p (−9.18), miR-151a-3p (−5.40), miR-361-3p (−4.30), miR-4298 (−3.88), miR-150-5p (−3.52), miR-199a-5p (−3.48), miR-374a-5p (−2.96) and miR-342-3p (−2.86). The top 100 significantly up-regulated miRNAs were listed in Online Resource 1. Only the top 100 dysregulated miRNAs were discussed in this study.

### Prediction of miRNA-targets in MM

Integrative analysis of significant differentially expressed miRNAs and mRNAs revealed 11 significantly expressed targeted genes (p < 0.05). They were *PCOLCE2, ASPM, CKAP2L, CCNA2, SHCBP1, RAD54L, NUF22, CTNNAL1, HKDC1, CYSLTR2*, and *RASGRF2*. When we associated the 11 targeted genes with the top 100 most significant dysregulated miRNAs, 5 putative target genes and 15 anti-correlated miRNAs were identified (Table 1). Theoretically, miRNAs are negative regulators for gene expression, the expression of a target mRNA is expected to be anti-correlated with miRNA expression (Lionetti et al. 2009). Therefore, only negative correlated miRNA-mRNA targets were discussed in this study.

**Table 1:** Significant negatively correlated miRNAs-mRNA expression networks identified in this study (p < 0.05)

Gene (fold change)	miRNA (fold change)
*RAD54L* (17.00)	miR-150-5p (−3.52)
*CCNA2* (19.69)	miR-150-5p (−3.52)
	miR-374a-5p (−2.96)
*CYSLTR2* (−7.35)	miR-125b-5p (21.91)
	miR-4698 (9.84)
	miR-1290 (17.32)
	miR-1183 (8.20)
	miR-9-5p (8.07)
	miR-4433-3p (7.90)
	miR-33a-5p (7.90)
	miR-4734 (7.89)
*HKDC1* (−9.53)	miR-1290 (17.32)
	miR-4698 (9.84)
	miR-4430 (9.34)
	miR-328 (8.22)
	miR-4763-5P (7.97)
*RASGRF2* (−5.52)	miR-125b-5p (8.22)
	miR-9-5p (8.07)
	mir-211-5p (7.91)
	miR-370 (7.90)

### Verification of microarray results by RT-qPCR

**Fig. 1:**
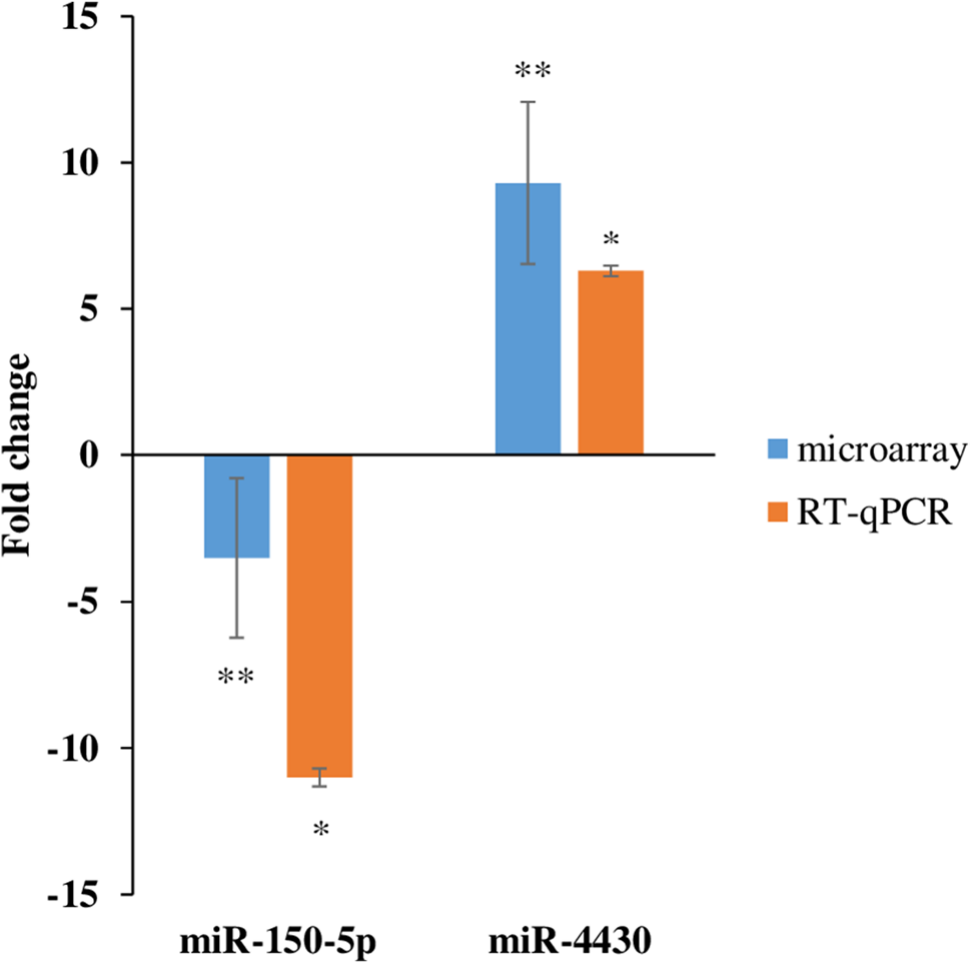
Verification of miRNA expression by RT-qPCR. The miR-150-5p and miR-4430 were down-regulated and up-regulated, respectively in MM compared to the controls. Importantly, the fold changes cannot directly compared between assays due to differences in calculation methods, but the general trend of up-regulation and down-regulation can be compared. Error bars represent the standard deviation of the mean (SD). *p < 0.001; **p < 0.05

Both miRNAs (miRNA-150-5p and miR-4430) were expressed at similar patterns as detected in microarray (p < 0.001) (Fig. 1).

## Discussion

### miRNA expression profiling

Previous studied showed that miRNA biogenesis in B cell malignancies are different with T cell malignancies in a way that the B-cell malignancies are more likely associated with a global increase in miRNA expression whereas T-cell malignancies with decrease in overall miRNA expression (Lawrie et al. 2008, 2009a, b; Zhang et al. 2009; Zhou et al. 2010). This is also true in our case. The vast majority of differentially expressed miRNAs identified in this study were over-expressed in MM compared to the controls. Several aberrant miRNAs, which play critical roles in survival, proliferation, migration, invasion and drug resistance in MM were revealed. A more detail summary of aberrant miRNAs and their function, target and clinical relevance in oncogenesis are listed in Table 2.

**Table 2:** Aberrant miRNAs and their function, target and clinical relevance in oncogenesis

miRNA	Fold change	Function/target/clinical relevance
miR-125b	21.9	Association with B cell maturation Malumbres et al. (2009)
		High expression resulted in down-regulation of *IRF4* and *Blimp1* Gururajan et al. (2010)
		Reduction of cell death in dexamethasone induced MM Murray et al. (2013)
		Transcriptional target of *p53* Pichiorri et al. (2010), Huang et al. (2012) and Zhang et al. (2015)
miR-148a	19.2	Up-regulation in plasma cells of MM and association with shorter progression free survival Huang et al. (2012)
miR-196b	12.5	Down-regulation increased *CCND2* expression and induced cell cycle at G1 to S phase Saki et al. (2014)
miR-21	10.9	Induction by *IL-6*/*STAT3* pathway upon adherence of bone marrow cells and bone marrow stromal cells Löffler et al. (2007)
		Over-expression inhibited apoptosis and increased drug resistance Wang et al. (2011)
		Association with early pathogenesis of MM Pichiorri et al. (2008)
miR-20a	9.2	Up-regulation by *c-MYC* over-expression Zhou et al. (2010)
		Aberration was associated with down-regulation of pro-apoptotic genes, *BIM* and *SOC-1* Anderson and Carrasco (2011)
		Up-regulation in plasma cells of MM and correlation with shorter progression free survival Chen et al. (2011) and Gao et al. (2012). Association with *CCND2* over-expression and promotion of cell cycle at G1 to S phase Saki et al. (2014)
miR-194-5p miR-215	8.4	Inhibition in cell migration and invasion by targeting
		*IGF1* and *IGF1R*
		Direct transcriptional target of *p53* Pichiorri et al. (2010) and Zhang et al. (2015)
		Down-regulation was associated with promoter hypermethylation, which would impair the *p53*/*MDM2* loop and promotion of MM development Pichiorri et al. (2010)
miR-330-3p	7.9	Over-expression in aggressive MM and association with shorter overall survival rate Lionetti et al. (2013)
miR-214	7.9	Down-regulation was caused by DNA methylation and resulted in inhibition of myeloma cell proliferation Gutiérrez et al. (2010)
		Target *PSMD10* and *ASF1B* Saki et al. (2014)
miR-150-5p	−3.5	Control B cell differentiation by targeting *c-Myb* Xiao et al. (2007) and Fernando et al. (2012)
		Promote cell growth, invasion and metastasis via interaction with *Mucin 4* Grammatikakis et al. (2013)
		Potential target of survivin Undi et al. (2013)
		Target tumour associated macrophages (*TAMs*) to induce *VEGF* production and tumour growth via angiogenesis Liu et al. (2013)
		Potential therapeutic target in MM Palagani et al. (2014)
miR-361-3p	−4.3	Association with t(11;14) translocation
		Target *PPP2R4*, the activation of *IL-6* signaling and resulted in increased cell growth and survival Lionetti et al. (2009)

Two important miRNAs involved in the IL6/STAT3 pathway were over-expressed in MM compared to the controls. They were miR-21 and miR-20a. The up-regulation of miR-21 is shown to facilitate the activation of IL6-JAK-STAT pathway and *STAT3*, which is a major mediator of growth, proliferation and survival of myeloma cells conferred by bone marrow microenvironment. The IL6/STAT3 activation enhances myeloma cell survival through the activation of anti-apoptotic genes, *Mcl-1, Bcl-XL* and *c-Myc* oncogene (Manier et al. 2012). It suggests that aberration in miR-21 contributes in the early onset of MM (Chi et al. 2011). Apart from that, miR-20a is a member of the miR-17-92 cluster—one of the well characterised class of oncogenic miRNAs. Its over-expression in MM is shown to inactivate apoptotic genes, *BIM* and *SOCS-1*, a negative regulator of IL-6/STAT3 pathway (Pichiorri et al. 2008).

Deletion/ mutation of *p53* gene play an important role in the oncogenesis of MM. The *p53* gene is deleted in only 5–10% of newly diagnosed MM cases but 40% of advanced MM (Gozzetti et al. 2014). The incidence of deletion/ mutation of *p53* increases as the stage of disease advances suggesting its critical role in disease progression (Gozzetti et al. 2014). Therefore, *p53* abnormality is associated with poor prognosis and lower survival rate in MM (Drach et al. 1998; Chng et al. 2007; Lodé et al. 2010). Apart from that, patient with *p53* abnormality has decreased sensitivity to standard therapy (Teoh and Chng 2014). Under normal condition, *p53* is expressed at low amount due to its continuous degradation by its negative regulator *MDM2* (Herrero et al. 2016). The *MDM2* is over-expressed in response to a variety of stress such as DNA damage, ribonucleotide depletion, nutritional starvation, hypoxia, and oncogene activation (Liu et al. 2014). Under stress condition, *p53* is stabilised through interruption of the MDM2-p53 interaction by mechanisms such as phosphorylation, acetylation, redistribution of protein complexes and modifications in the subcellular localisation, which resulted in p53 pathway activation (Feng and Levine 2010; Vousden and Prives 2009). Activation of p53 pathway mediated tumour suppressive mechanisms in its downstream signaling. The loss of p53 protein turning off the activity of p53 networks, and resulted in suppression of cell cycle arrest genes (*p21, GADD45A* and *14-3-3*σ), apoptosis genes (*Bax, PUMA, Noxa* and *Bid*) and senescence (*p21*) (Sax et al. 2002; Herrero et al. 2016). Loss of p53 function also promotes angiogenesis (*TSP1* and *maspin*) (Herrero et al. 2016). Besides that, *p53* disruption also affects cell metabolism, autophagy, necrosis, anti-oxidant defense and microRNA expression (Hager and Gu 2014; Liu et al. 2014). Altogether, they facilitate the proliferation and pro-longed survival of MM cells. The miR-125b, miR-194 and miR-215 are potential therapeutic targets in MM, which are related to p53 pathway (Pichiorri et al. 2010). These miRNAs are transcriptionally activated by *p53* and able to form positive feedback loop with *p53* to help to activate the gene under stress condition (Bi and Chng 2014). The miR-125b, miR-194 and miR-215 also capable of inhibit cancer cell proliferation through promoting the p53-mediated apoptosis, cell cycle arrest and senescence (Zhang et al. 2015). These miRNAs are commonly down-regulated in MM (Pichiorri et al. 2010). However, instead of under-expressed, they were over-expressed in this study. This could be explained by the complexity of regulations and functions of miRNAs in cancers. Research found that a single miRNA can act as oncomir or tumour suppressor simultaneously (Li et al. 2012). For example, miR-196b can target either tumour suppressor, *FAS* or oncogene, *HOXA9*/*MEIS1* in leukaemia (Li et al. 2012). When miR-196b was over-expressed in leukaemia cells, it represses the function of *FAS* and at the same time promotes cell proliferation and inhibits apoptosis via increases expression of *HOXA9*/*MEIS1*. Apart from that, miRNAs regulate gene expression at the post-transcriptional level and at the same time their expression are regulated by transcription factors (Wang et al. 2010). Moreover, other studied showed that the expression of miRNAs is not only regulated at transcriptional level but also at the post-transcriptional level during the downstream processing stages (Siomi and Siomi 2010). All of these findings imply that the expression of the miRNA in oncogenesis is varied depending on the potential influences of the different biological and cellular contexts (Nam et al. 2014).

### miRNA-target prediction

The miRNA-mRNA enrichment analysis revealed that *CCNA2, CYSLTR2, RASGRF2* and *HKDC1* were targeted by more than one miRNAs (except for *RAD54L*) (Table 1). Out of 15 putative miRNAs identified in this study, only a few of them are concomitant with cancers. They are miR-150, miR-125b, miR-33a, miR-9 and miR-211. Interestingly, miR-150 and miR-125b are closely related to B cell differentiation and therefore highlighted their critical roles in myelomagenesis. The *c-Myb, Mucin 4, TAMs* and survivin/*BIRC5* are a few potential targets of miR-150 (Xiao et al. 2007; Fernando et al. 2012; Grammatikakis et al. 2013; Liu et al. 2013; Undi et al. 2013). So far, there is no evidence showing the biological relationship between miR-150 and *RAD54L* or *CCNA2*. For the first time, our findings exhibited the possible roles of miR-150 in regulating two important cell cycle associated genes, *RAD54L* and *CCNA2*. The *RAD54L* is involved in DNA double strand break repair and chromatin remodeling in G1/ S-transition via homologous recombination (Mjelle et al. 2015). Its misrepaired would cause various mutations, deletions and oncogenic translocations in human cells (Agarwal et al. 2011; Mjelle et al. 2015). Elevated expression of *RAD54L* was identified in colon and breast cancer, lymphoma and meningioma (Leone et al. 2003). We revealed for the first time that *RAD54L* was over-expressed in MM and its aberrant expression might be caused by down-regulation of miR-150. Another putative target of miR-150, the *CCNA2* gene is a well-established cell cycle control gene, which is a genetic marker for prognostic and outcome prediction in MM and other cancers (García-Escudero et al. 2010).

Aberrant expression of miR-125b was implicated in various cancers such as colon, prostate, and lymphoma (Jacinto et al. 2007; Mahapatra et al. 2012). The *IRF4, Blimp1* and *p53* were identified as its possible targets (Table 2). Our integrative analysis of miRNA-mRNA expression data predicted new potential targets of miR-125b, namely *RASGRF2* and *CYSLTR2*. Low expression of *RASGRF2* has been described in lymphoma, lung cancer, cancer cell lines and primary tumour but not in MM (Chen et al. 2006). The RASGRF2 is a RAS signaling protein involved in regulating conversion of active or inactive forms of RAS protein (Chen et al. 2006). RAS protein is an important component in signal transduction pathway as it activates intracellular pathways that affect the biological processes related to cell proliferation, survival, and motility (Agarwal et al. 2011). We suggests that disruption of normal signaling pathways through under-expression of *RASGRF2* might facilitate cancerous cell growth and invasion. Besides *RASGRF2, CYSLTR2* is another potential target of miR-125b identified in this study. The role of *CYSLTR2* in myelomagenesis is still unknown although its dysregulation has been shown to facilitate cell proliferation and migration of colon cancer cells via CysLT signaling (Bengtsson et al. 2013).

Other potential miRNAs such as dysregulation of miR-33a, miR-9 and miR-211 have never been reported in association with MM transformation and disease progression. However, they have been shown to play crucial roles in other cancers such as breast, ovarian, melanoma, and glioblastoma (Ibrahim et al. 2011; Blandino et al. 2012; Wang et al. 2013; Xia et al. 2015).

This study has limitation due to the used of small sample size. In addition, CD138+ plasma cells enrichment was not performed due to the limited capacity of bone marrow samples received from the patients. Our future study aims to confirm the microarray findings with larger sample size, possibly with CD138+ purified plasma cells from the patients. Besides that, the miRNA-targets identified in this study is predicted based on the integrative analysis of miRNA and mRNA expression profiles of the matched samples, the biological relevance of the miRNA-targets need to be further verified by using dual-luciferase reporter assay system.

## Conclusions

Our present study revealed potential miRNAs and miRNA-target underlying the molecular pathogenesis of MM. One of the most significant findings are the identification of new possible targets for two B cell related miRNAs, miR-150 and miR-125b. Our findings predicted that miR-150 might be negative regulator for two critical cell cycle control genes, *RAD54L* and *CCNA2*, whereas miR-125b potentially target RAS and CysLT signaling proteins, namely RASGRF2 and CYSLTR2. The miRNA and miRNA-target identified in this study might be important therapeutic markers in MM, which is worthy for further investigation.

## Electronic supplementary material

Below is the link to the electronic supplementary material.

Supplementary material 1 (DOCX 16 KB)
