# Supplementary material for: MicroRNA expression patterns and target prediction in multiple myeloma development and malignancy
Source: Genes Genomics. 2017 Feb 9;39(5):533–40. doi: 10.1007/s13258-017-0518-7 (PMC5387019; doi:10.1007/s13258-017-0518-7)
Supplement: Supplementary file 1 — Supplementary material 1 (DOCX 16 KB) [file 13258_2017_518_MOESM1_ESM.docx]

Top 100 up-regulated miRNAs in MM compared to normal controls by >2.0 fold change at p<0.05.

| **No** | **systematic name** | **Fold Change (Multiple Myeloma vs Normal)** | **mirbase accession No** |
| --- | --- | --- | --- |
| 1 | hsa-miR-193b-3p | 72.696 | MIMAT0002819 |
| 2 | hsa-miR-183-5p | 42.81074 | MIMAT0000261 |
| 3 | hsa-miR-96-5p | 33.432415 | MIMAT0000095 |
| 4 | hsa-miR-630 | 30.826603 | MIMAT0003299 |
| 5 | hsa-miR-551b-3p | 28.938314 | MIMAT0003233 |
| 6 | hsa-miR-125b-5p | 21.910614 | MIMAT0000423 |
| 7 | hsa-miR-148a-3p | 19.154634 | MIMAT0000243 |
| 8 | hsa-miR-99a-5p | 18.97416 | MIMAT0000097 |
| 9 | hsa-miR-5703 | 18.140188 | MIMAT0022496 |
| 10 | hsa-miR-9-3p | 17.780647 | MIMAT0000442 |
| 11 | hsa-miR-1290 | 17.324305 | MIMAT0005880 |
| 12 | hsa-miR-1228-3p | 16.917711 | MIMAT0005583 |
| 13 | hsa-miR-6073 | 16.259237 | MIMAT0023698 |
| 14 | hsa-miR-4651 | 15.470672 | MIMAT0019715 |
| 15 | hsa-miR-4449 | 14.707131 | MIMAT0018968 |
| 16 | hsa-miR-196b-5p | 12.451211 | MIMAT0001080 |
| 17 | hsa-miR-143-3p | 12.203119 | MIMAT0000435 |
| 18 | hsa-miR-4778-5p | 11.505524 | MIMAT0019936 |
| 19 | hsa-miR-3188 | 11.321307 | MIMAT0015070 |
| 20 | hsa-miR-1234-3p | 11.275728 | MIMAT0005589 |
| 21 | hsa-miR-4446-3p | 11.0353155 | MIMAT0018965 |
| 22 | hsa-miR-3648 | 10.888356 | MIMAT0018068 |
| 23 | hsa-miR-210 | 10.8686695 | MIMAT0000267 |
| 24 | hsa-miR-21-3p | 10.865276 | MIMAT0004494 |
| 25 | hsa-miR-718 | 10.6935005 | MIMAT0012735 |
| 26 | hsa-miR-4484 | 10.66886 | MIMAT0019018 |
| 27 | hsa-miR-765 | 10.617614 | MIMAT0003945 |
| 28 | hsa-miR-3682-3p | 10.019291 | MIMAT0018110 |
| 29 | hsa-miR-4698 | 9.836284 | MIMAT0019793 |
| 30 | hsa-miR-1246 | 9.541015 | MIMAT0005898 |
| 31 | hsa-miR-1233-1-5p | 9.489333 | MIMAT0022943 |
| 32 | hsa-miR-422a | 9.459081 | MIMAT0001339 |
| 33 | hsa-miR-4430 | 9.340585 | MIMAT0018945 |
| 34 | hsa-miR-550a-3p | 9.284138 | MIMAT0003257 |
| 35 | hsa-miR-1469 | 9.275376 | MIMAT0007347 |
| 36 | hsa-miR-4685-5p | 9.191523 | MIMAT0019771 |
| 37 | hsa-miR-497-5p | 9.180842 | MIMAT0002820 |
| 38 | hsa-miR-20a-3p | 9.175086 | MIMAT0004493 |
| 39 | hsa-miR-431-3p | 9.139491 | MIMAT0004757 |
| 40 | hsa-miR-193b-5p | 9.138991 | MIMAT0004767 |
| 41 | hsa-miR-598 | 8.991602 | MIMAT0003266 |
| 42 | hsa-miR-6512-5p | 8.982444 | MIMAT0025480 |
| 43 | hsa-miR-196a-5p | 8.972707 | MIMAT0000226 |
| 44 | hsa-miR-132-3p | 8.964459 | MIMAT0000426 |
| 45 | hsa-miR-23a-5p | 8.565206 | MIMAT0004496 |
| 46 | hsa-miR-627 | 8.497789 | MIMAT0003296 |
| 47 | hsa-miR-940 | 8.402196 | MIMAT0004983 |
| 48 | hsa-miR-205-5p | 8.398831 | MIMAT0000266 |
| 49 | hsa-miR-194-5p | 8.380732 | MIMAT0000460 |
| 50 | hsa-miR-215 | 8.379484 | MIMAT0000272 |
| 51 | hsa-miR-100-5p | 8.295616 | MIMAT0000098 |
| 52 | hsa-miR-4538 | 8.249183 | MIMAT0019081 |
| 53 | hsa-miR-328 | 8.221823 | MIMAT0000752 |
| 54 | hsa-miR-4690-5p | 8.220384 | MIMAT0019779 |
| 55 | hsa-miR-340-5p | 8.207941 | MIMAT0004692 |
| 56 | hsa-miR-1183 | 8.20255 | MIMAT0005828 |
| 57 | hsa-miR-4701-5p | 8.157882 | MIMAT0019798 |
| 58 | hsa-miR-4522 | 8.153399 | MIMAT0019060 |
| 59 | hsa-miR-4436b-5p | 8.149684 | MIMAT0019940 |
| 60 | hsa-miR-550a-3-5p | 8.132994 | MIMAT0020925 |
| 61 | hsa-miR-4745-5p | 8.128381 | MIMAT0019878 |
| 62 | hsa-miR-885-5p | 8.117832 | MIMAT0004947 |
| 63 | hsa-miR-10b-3p | 8.104628 | MIMAT0004556 |
| 64 | hsa-miR-203a | 8.083766 | MIMAT0000264 |
| 65 | hsa-miR-129-1-3p | 8.07458 | MIMAT0004548 |
| 66 | hsa-miR-9-5p | 8.074543 | MIMAT0000441 |
| 67 | hsa-miR-4530 | 8.069372 | MIMAT0019069 |
| 68 | hsa-miR-516a-5p | 8.051895 | MIMAT0004770 |
| 69 | hsa-miR-542-3p | 8.021954 | MIMAT0003389 |
| 70 | hsa-miR-4649-3p | 8.006126 | MIMAT0019712 |
| 71 | hsa-miR-4792 | 8.002802 | MIMAT0019964 |
| 72 | hsa-miR-10b-5p | 7.994181 | MIMAT0000254 |
| 73 | hsa-miR-7-1-3p | 7.986075 | MIMAT0004553 |
| 74 | hsa-miR-4763-5p | 7.9657655 | MIMAT0019912 |
| 75 | hsa-miR-4767 | 7.959038 | MIMAT0019919 |
| 76 | hsa-miR-181d | 7.953306 | MIMAT0002821 |
| 77 | hsa-miR-424-3p | 7.9429507 | MIMAT0004749 |
| 78 | hsa-miR-30a-5p | 7.9296923 | MIMAT0000087 |
| 79 | hsa-miR-4743-5p | 7.9248753 | MIMAT0019874 |
| 80 | hsa-miR-200b-3p | 7.924423 | MIMAT0000318 |
| 81 | hsa-miR-3617-3p | 7.9185796 | MIMAT0022966 |
| 82 | hsa-miR-4697-3p | 7.918378 | MIMAT0019792 |
| 83 | hsa-miR-4695-3p | 7.9177628 | MIMAT0019789 |
| 84 | hsa-miR-34b-5p | 7.908678 | MIMAT0000685 |
| 85 | hsa-miR-211-5p | 7.9085317 | MIMAT0000268 |
| 86 | hsa-miR-4728-3p | 7.90797 | MIMAT0019850 |
| 87 | hsa-miR-518e-5p | 7.9050035 | MIMAT0005450 |
| 88 | hsa-miR-650 | 7.900557 | MIMAT0003320 |
| 89 | hsa-miR-370 | 7.897305 | MIMAT0000722 |
| 90 | hsa-miR-4433-3p | 7.896072 | MIMAT0018949 |
| 91 | hsa-miR-6075 | 7.896021 | MIMAT0023700 |
| 92 | hsa-miR-33a-5p | 7.895879 | MIMAT0000091 |
| 93 | hsa-miR-664a-5p | 7.8958526 | MIMAT0005948 |
| 94 | hsa-miR-4695-5p | 7.8933973 | MIMAT0019788 |
| 95 | hsa-miR-2276 | 7.8887444 | MIMAT0011775 |
| 96 | hsa-miR-4734 | 7.8881354 | MIMAT0019859 |
| 97 | hsa-miR-6069 | 7.8876023 | MIMAT0023694 |
| 98 | hsa-miR-214-3p | 7.8817987 | MIMAT0000271 |
| 99 | hsa-miR-4417 | 7.881361 | MIMAT0018929 |
| 100 | hsa-miR-330-3p | 7.861473 | MIMAT0000751 |
